# Is the Golden hour important? Looking at disability and health-related quality of life in a Portuguese trauma registry

**DOI:** 10.1186/cc13253

**Published:** 2014-03-17

**Authors:** J Estilita, CC Dias, A Costa-Pereira, C Granja, I Arag±o, L Orwelius

**Affiliations:** 1 DEICM, Algarve Hospital Centre, Portugal; 2 University of Algarve, Portugal; 3 CINTESIS, FMUP, Porto, Portugal; 4 FMUP, Porto, Portugal; 5 Linköping University, Linköping, Sweden

## Introduction

The Golden hour (GH) complies the concept that definitive trauma care must be initiated within 60 minutes in order to improve outcome, but evidence is conflicting [1][2]. A variety of injury and health loss scores are used in post-impact care for assessing the injury severity, probability of survival and long-term loss of health [3]. The aim of this study is to establish a relationship between the concept of GH and disability or health-related quality of life (HRQoL) in trauma patients and to compare injury scores and health loss scores in this population.

## Methods

We analyzed patients from a trauma registry of a 700-bed university hospital between January 2003 and December 2007 who were alive 6 months after injury. A follow-up interview with the EQ-5D questionnaire was conducted. Data included patient demographics, type of injury, ISS, RTS and TRISS scores, ICU and hospital length of stay (LOS), mortality and EQ-5D domains.

## Results

There were 78% (*n *= 589) males and the average age was 44 ± 20 years. Most trauma cases originated from metropolitan areas and 91% (*n *= 688) of patients were managed within the GH. There was an association of mortality with older age and penetrating trauma. Mortality was higher for those with shorter length of stay. Those treated within the GH were less likely to die. In the HRQoL analysis, over one-half of the patients had moderate to severe problems in the Usual Activities domain. The median EQ-5D Index was 0.66 (0.47 to 0.98). There was no association of GH with HRQoL domains 6 months after discharge from the ICU. For the TRISS there was a lower probability of survival for the patients with severe problems in the physical health domains. The same results were obtained for association with age and LOS.

## Conclusion

The GH may be important as a survival outcome but not as a HRQoL outcome. Patients with severe problems in EQ-5D physical health domains showed lower probability of survival, were older and had a longer ICU LOS.
